# Effect of cigarette smoke on counts of immunoreactive cells to eotaxin-1 and eosinophils on the nasal mucosa in young patients with perennial allergic rhinitis^☆^

**DOI:** 10.1016/j.bjorl.2016.04.011

**Published:** 2016-05-19

**Authors:** Bertha Beatriz Montaño-Velázquez, Eulalia Beatriz Flores-Rojas, Francisco Javier García-Vázquez, Silvio Jurado-Hernandez, Marco Antonio Venancio Hernández, Angélica Kathya Alanis Flores, Kathrine Jáuregui-Renaud

**Affiliations:** a Instituto Mexicano del Seguro Social, Centro Médico Nacional La Raza, Service of Otorhinolaryngology, Mexico City, Mexico; b Instituto Nacional de Pediatría, Molecular Pathology Laboratory, Department of Pathology Anatomy, Mexico City, Mexico; c Instituto Mexicano del Seguro Social, CMN La Raza, Hospital de Especialidades, Service of Immunology and Allergy, Mexico City, Mexico; d Instituto Mexicano del Seguro Social, Centro Médico Nacional sXXI, P.B. Edificio C Salud en el Trabajo, Medical Research Unit in Otoneurology, Mexico City, Mexico

**Keywords:** Rhinitis, Allergic, Tobacco, Chemokine CCL11, Eosinophils, Rinite, Alérgico, Tabaco, Quimiocina CCL11, Eosinófilos

## Abstract

**Introduction:**

In teenagers with perennial allergic rhinitis, exposure to tobacco cigarette smoke increases the count of eosinophils in the nasal mucosa; the recruitment of eosinophils arises from the combined action of a number of cellular and molecular signals, including eotaxin.

**Objective:**

To assess the effect of exposure to tobacco cigarette smoke on the count of immunoreactive cells to eotaxin-1 and eosinophils on the nasal mucosa of children and teenagers with perennial allergic rhinitis.

**Methods:**

In a cross-sectional study, forty-four patients were evaluated (aged 7–19 years old): 22 with and 22 with no exposure to tobacco cigarette smoke. After replying to 2 validated questionnaires, on Asthma and Allergies in Childhood and on the severity of nasal symptoms, nasal mucosal samples were obtained by scraping the middle one-third of the inferior turbinates. Then counts of immunoreactive cells to eotaxin-1 and eosinophils were assessed by immunohistochemistry.

**Results:**

Patients with exposure to tobacco cigarette smoke showed higher cell counts of both eotaxin-1 and eosinophils than patients with no exposure to the smoke, with no correlation between the two variables. However, both counts, of eotaxin-1 and eosinophils, were related to the cotinine/creatinine ratio.

**Conclusions:**

Exposure to tobacco cigarette smoke can increase eotaxin-1 and the count of eosinophils in the nasal mucosa of young patients with perennial allergic rhinitis.

## Resumo

**Introdução:**

Em adolescentes com rinite alérgica perene, a exposição à fumaça do cigarro de tabaco aumenta a contagem de eosinófilos na mucosa nasal. O recrutamento de eosinófilos surge da ação combinada de alguns sinais celulares e moleculares, incluindo a eotaxina.

**Objetivo:**

Avaliar o efeito da exposição à fumaça do cigarro de tabaco na contagem de células imunorreativas a eotaxina-1 e eosinófilos na mucosa nasal de crianças e adolescentes com rinite alérgica perene.

**Método:**

Em um estudo transversal, 44 pacientes foram avaliados (entre sete e 19 anos): 22 com e 22 sem exposição à fumaça do cigarro de tabaco. Depois de responder a dois questionários validados, sobre asma e alergias na infância e sobre a gravidade dos sintomas nasais, as amostras de mucosa nasal foram obtidas por meio de raspagem do terço médio das conchas inferiores. Em seguida, as contagens de células imunorreativas para eotaxina-1 e eosinófilos foram avaliadas por imuno-histoquímica.

**Resultados:**

Os pacientes com exposição à fumaça do cigarro de tabaco apresentaram contagens de células mais elevadas tanto para eotaxina-1 como para eosinófilos em comparação com os pacientes sem exposição à fumaça, sem correlação entre as duas variáveis. No entanto, ambas as contagens, de eotaxina-1 e eosinófilos, foram relacionadas com a razão cotinina/creatinina.

**Conclusões:**

A exposição à fumaça do cigarro de tabaco pode aumentar a eotaxina-1 e a contagem de eosinófilos na mucosa nasal de pacientes jovens com rinite alérgica perene.

## Introduction

Allergic diseases, such as allergic asthma, allergic rhinitis and atopic dermatitis are characterized by an increased number of eosinophils in the circulating blood, and degranulation in the target tissue is considered the major pathogenic event.^1^ The recruitment of eosinophils arises from the combined action of a number of cellular and molecular signals, including eotaxin.^2^, ^3^ Eotaxin-1 binds with high affinity to CC chemokine receptor 3, which is expressed by a variety of inflammatory cells.^4^, ^5^, ^6^ Blocking eotaxin or CCR3 has been proposed as a new approach to allergy immunotherapy.^7^, ^8^ However, more information about the interaction between the ligands and their receptors is required.

Allergic rhinitis is a common condition affecting people of all ages, with peak lifetime prevalence occurring in teenagers.^9^ It has been shown that, in adult patients with allergic rhinitis, nasal allergen challenge may led to parallel increases of the count of eosinophils and eotaxin levels in nasal lavage fluid, with a strong correlation between the two variables.^10^ In natural conditions of disease, without nasal challenge, compared with controls, eotaxin-1 concentration in nasal lavage fluid from patients with allergic rhinitis is increased in both, the perennial and seasonal forms, and it is related to the percentage of lavage eosinophils and the severity of symptom expression.^11^

Children exposed to environmental tobacco smoke have an increased risk of developing respiratory-tract illnesses. Experiments in murine models show that tobacco smoke can elicit a rapid and prolonged exaggerated immune response.^12^, ^13^ In humans, the effects of tobacco smoke on the upper respiratory airways include the recruitment and activation of inflammatory cells.^14^ In teenagers with perennial allergic rhinitis, patients exposed to tobacco cigarette smoke, compared with those with no exposure, may have an increased count of eosinophils in the nasal mucosa.^15^ In patients with asthma smoking increases eotaxin levels.^16^

The purpose of this study was to assess the influence of exposure to tobacco cigarette smoke on the counts of immunoreactive cells to eotaxin-1 and eosinophils in the nasal mucosa of children and teenagers with perennial allergic rhinitis.

## Methods

### Ethical considerations

The protocol was approved by the Local Research and Ethics Committee, and informed consent was obtained from all patients and their parents.

### Participants

Forty-four patients with perennial allergic rhinitis participated in the study, all living within the same city area of Mexico City. Inclusion in the study was considered consecutively when perennial allergic rhinitis was diagnosed for the first time and patients had no evidence of infection, sinusitis, otitis media, nasal polyps, anatomical abnormality, systemic disease, lung disease, asthma, atopic dermatitis, seasonal allergic rhinitis or pregnancy; neither they have used immunotherapy, corticosteroids (nasal or systemic), cromolin, anti-inflammatory treatment or antileukotrienes within 3 months prior to participating in the study.

According to the exposure to tobacco cigarette smoke, they were classified in two groups, with a similar age, weight, body mass index and time of clinical evolution (Table 1):

**Table 1:** Characteristics of 44 patients with perennial allergic rhinitis: 22 with and 22 with no exposure to cigarette tobacco smoke.

Variables (mean ± SD)	Passive exposure (*n* = 22)	No exposure (*n* = 22)	*p* ≤ 0.05
Age (years)	12.09 ± 3.0	11.9 ± 3.2	–
Weight (kg)	47.09 ± 14.99	44.46 ± 15.48	–
Height (m)	1.48 ± 0.13	1.45 ± 0.16	–
Body mass index	20.98 (4.6)	20.44 (3.43)	–
Time of clinical evolution (years)	3.6 ± 2.1	4.4 ± 3.3	–
Urine cotinine/creatinine ratio (ng/mg)	25.98 ± 3.7	11.21 ± 2.47	≤0.01
Eotaxin-1 (immunoreactive cells per square millimeter)	194 ± 178	4 ± 7	≤0.01
Eosinophils count (immunoreactive cells per square millimeter)	198 ± 264	18 ± 40	≤0.01

Allergens	Frequency (*n*)	Frequency (*n*)	
*Dermatophagoides* sp.	40% (9)	50% (11)	–
House mites	45% (10)	36% (8)	–
Cockroach	32% (7)	36% (8)	–

Nasal symptoms	Frequency (*n*)	Frequency (*n*)	
Obstruction	90% (20)	86.36% (19)	–
Rhinorrhea	95.45% (21)	95.45% (21)	–
Itching	81.81% (18)	81.81% (18)	–
Sneezing	86.36% (19)	68.18% (15)	–
Total score (median and interval)	6 (4–8)	6 (4–8)	–

Group I – 22 patients exposed to tobacco cigarette smoke. They were aged 7–19 years (mean ± standard deviation 12 ± 3 years), 12 were males and 10 were females.

Group II – 22 patients not exposed to tobacco cigarette smoke. They were aged 7–17 years (11.9 ± 3.2 years), 15 were males and 7 were females.

### Procedures

Exposure or no exposure to tobacco cigarette smoke was determined by means of The Global Youth Tobacco Survey,^17^ and by urinary cotinine/creatinine ratio.^18^ On the same day that participants replied to the questionnaire, their urine was collected to assess their cotinine/creatinine ratio. Only when the two evaluations were consistent, patients were included in the study. The cotinine/creatinine ratio was assessed by solid-phase competitive chemiluminescent immunoassay for cotinine (Metabolites of Nicotine, DPC France; Immulite 1000, DPC, NJ, USA) and colorimetric Jaffé method for creatinine (Clinical Chemistry IL Test™, Spinreac, Saint Esteve de Bas, Spain; Express Plus, Bayer, Tarrytown, NY, USA). A cut-off value of 21.8 ng/mg of cotinine/creatinine ratio was used to identify exposure to tobacco smoke.^18^

After a clinical evaluation was performed, patients replied to the short version of the questionnaire from the “International Study of Asthma and Allergies in Childhood”^9^ and a validated questionnaire of the severity of nasal symptoms in children with perennial allergic rhinitis.^19^ The symptoms evaluated were: congestion, sneezing, itching and rhinorrhea. The severity of each symptom was rated by the patient as absent (0), mild (1), moderate (2) or severe (3). Total symptom score was calculated as the sum of each symptom score (maximum = 12).^19^ In children and teenagers with perennial allergic rhinitis, this questionnaire has shown a consistency of 0.89 and repeatability of 96%, with a repeatability coefficient of 2.^20^

Nasal mucosal samples were obtained by scraping the middle one-third of the inferior turbinates (Rhinoprobe Arlington Scientific Inc., Arlington, TX, USA) and were stained with Wright-Giemsa stain. All samples were analyzed by immunohistochemistry, by two independent reviewers, on the slides of ten calibrated fields that were randomly selected (Leica, DM750, 40×), to determine the counts of immunoreactive cells to eotaxin-1 and eosinophils per squared millimeter, using rabbit monoclonal antibody chemokine (C—C motif) ligand 11 (Eotaxin EPR5825; Gene-Tex Irving, CA, USA) and mouse monoclonal antibodies (mouse mayor basic protein BMK13, 1:25; Chemicon International, Temecula, CA, USA) respectively.

### Statistical analysis

After Kolmogorov Smirnov test, statistical analysis was performed according to data distribution using *t* test, Pearson's correlation coefficient and Analysis of Covariance, values of *p* ≤ 0.05 were considered significant.

## Results

### Clinical characteristics of the patients

The general characteristics of the patients are described in Table 1. Among the patients with exposure to tobacco smoke (Group I), 21 patients reported only passive exposure and one patient reported both, passive and active exposure. In the two groups, the number of positive allergens during prick testing (AllerStand, Mexico City; IRC guidelines, 1994) was from 1 to 13 (median 3) and the most frequent allergen was *Dermatophagoides* sp. (40% in Group I vs. 50% in Group II).

The frequency and severity of the nasal symptoms is described in Table 1. There was no difference between groups either on the frequency of each symptom or the total score (median 6, in the 2 Groups). The most frequent symptom was rhinorrhea, which was reported by 95% of the patients in the two groups.

#### Immunoreactive cell counts of eotaxin-1 and eosinophils

**Figure 1:**
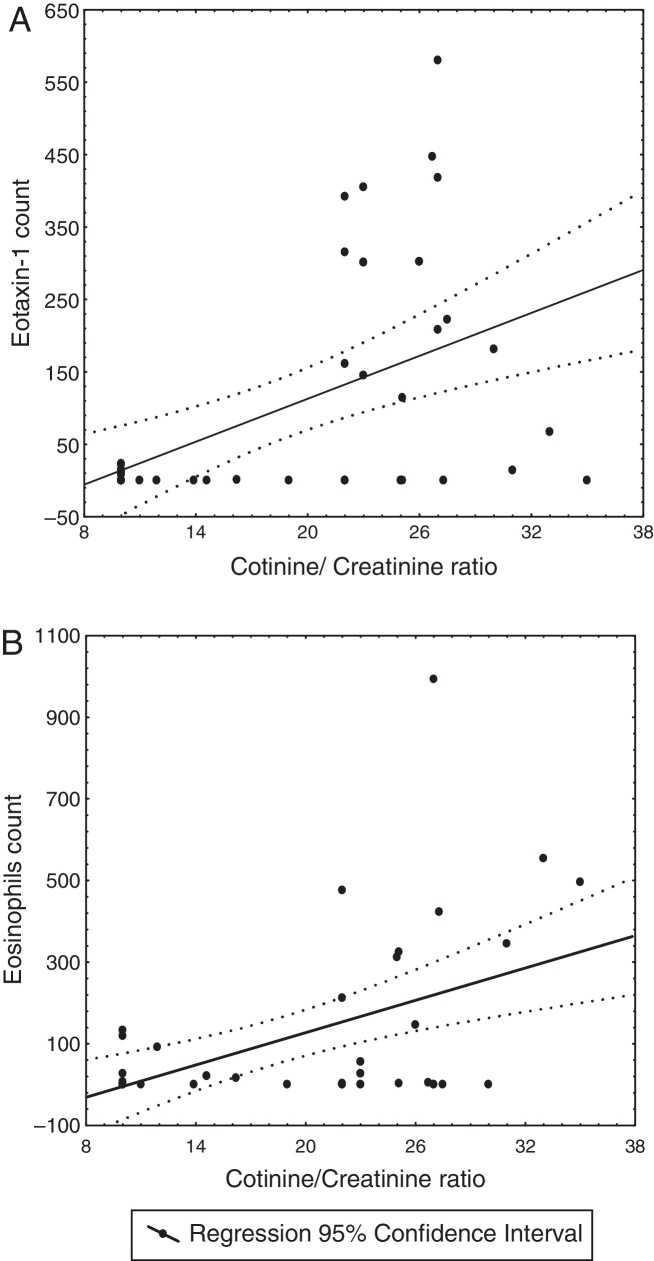
Linear relationship between the urine cotinine/creatinine ratio and (A) the count of immunoreactive cells to eotaxin-1 and (B) the count of eosinophils, in the nasal mucosa of 44 patients with perennial allergic rhinitis.

Patients with exposure to tobacco cigarette smoke had higher counts of immunoreactive cells in the nasal mucosa for both eotaxin-1 and eosinophils than patients with no exposure (*t* Student, *p* < 0.05) (Table 1). Although the two variables showed no linear relationship between them, in the whole group, the cell counts of both eotaxine-1 and eosinophils were related to the urine cotinine/creatinine ratio (Pearson's *r* = 0.51 and 0.50 respectively, *p* < 0.001) (Fig. 1).

Analysis of Covariance showed that the relationship between the cell count of eotaxin-1 and the urine cotinine/creatinine ratio was independent from the age, the gender and the body mass index of the patients (*p* > 0.05), while the relationship between the cell count of eosinophils and the urine cotinine/creatinine ratio was related to the age (*β* = −0.6, 95% CI 0.89–0.31) and to the body mass index (*β* = 0.44, 95% CI 0.15–0.74) (whole model *R* = 0.7, *p* < 0.001).

## Discussion

The results of this study show evidence that compared to children and teenagers with no exposure to tobacco cigarette smoke, those with perennial allergic rhinitis who were exposed to tobacco smoke may have increased immunoreactive cell counts in their nasal mucosa to both eotaxin-1 and eosinophils, which are related to their urine cotinine/creatinine ratio.

In animal models and humans, several effects of exposure to tobacco smoke on the immune response have been described,^21^, ^22^, ^23^, ^24^ including modified blood counts of eosinophils and monocytes.^25^ However, there is a lack of studies assessing the effect of tobacco smoke exposure on eotaxin-1 in patients with allergic rhinitis. This study shows that even passive exposure to tobacco smoke may increase immunoreactive cell counts to eotaxin-1 in the nasal mucosa, which are linearly related to the exposure. Then, in patients with allergic rhinitis, assessment and control of the exposure to tobacco smoke may contribute to prevent further damage to the nasal mucosa.

Interestingly, immunoreactive cell counts of eotaxin-1 were not related to the counts of eosinophils or to the characteristics of the subjects. This finding could be explained because eotaxin-1 binds with high affinity to CC chemokine receptor 3, which is expressed by a variety of inflammatory cells, including eosinophils, mast cells, basophils, and T helper type 2 lymphocytes.^4^, ^5^, ^6^ Then, CCR3-eotaxin are expressed not only in cells implicated in activation or migration of eosinophils but also in various other cells involved in allergic inflammation.^26^, ^27^

The relationship between the count of eosinophils and the body mass index, observed in the whole group of patients, is in agreement with previous reports showing a proportional relationship between the body mass index and allergic diseases of the airways.^15^, ^28^, ^29^ Although further studies are needed to understand its meaning.

The finding of a similar frequency/severity of nasal symptoms in patients with allergic rhinitis exposed or not to tobacco smoke, might have been related to the age of the participants. Evidence support that children and teenagers with perennial rhinitis frequently under-report their nasal symptoms.^30^, ^31^ Additionally, the study setting may have been a confounder; all participants were living in one of the most polluted cities in the world. Since the two groups were similarly exposed to pollution, they were comparable, but environmental factors may have interfered with their symptoms. Several between- and within-country associations between environmental factors and persistent rhinitis symptom prevalence have been reported.^32^, ^33^ In Peru^34^ and in Brazil,^35^ higher current asthma and rhinitis symptoms have been observed in urban teenagers as compared to those living in rural villages.

Evidence supports that sex hormones are likely to play an important role in the development and outcome of the allergic immune response.^36^ Although, this study was not designed to explore it, the similar age and male/female ratio of the two groups allowed comparisons between them, while controlling for a possible influence of these factors on the results.

The main limitations of this study are the design and the sample size. The cross-sectional design prevent us to discuss any causal relationship; and the sample size allowed us to identify only the most evident differences, without denying other possible relationships among the variables of the study. Although, the selection criteria were useful to control the influence of the main confounders (like concurrent inflammatory diseases and immunotherapy), a possible influence on the results of other potential confounders, like food intake and the respiratory exposure to other substances, cannot be excluded.

## Conclusion

Exposure to tobacco cigarette smoke can increase eotaxin-1 and the count of eosinophils in the nasal mucosa of young patients with perennial allergic rhinitis.

## Conflicts of interest

The authors declare no conflicts of interest.
